# Chromosome 1 variants associated with decreased HIV set-point viral load correlate with PRKAB2 expression changes

**DOI:** 10.3389/fgene.2025.1551171

**Published:** 2025-03-06

**Authors:** Riley H. Tough, Paul J. McLaren

**Affiliations:** ^1^ Sexually Transmitted and Blood-Borne Infections Division, National Microbiology Laboratories, Public Health Agency of Canada, Winnipeg, MB, Canada; ^2^ Department of Medical Microbiology and Infectious Diseases, University of Manitoba, Winnipeg, MB, Canada

**Keywords:** genome-wide association studies, bioinformatics, PRKAB2, RNA-sequencing, HIV - human immunodeficiency virus

## Abstract

A previous study investigated a genomic region on chromosome 1 associated with reduced human immunodeficiency virus type 1 (HIV) set-point viral load, implicating *CHD1L* as a novel HIV inhibitory factor. However, given that regulatory variants can influence expression of multiple nearby genes, further work is necessary to determine the impact of genetic variants on other genes in the region. This study evaluates the potential for genetic regulation of *PRKAB2*, a gene located upstream of *CHD1L* and encoding the β2 regulatory subunit of the AMPK complex, and for downstream impacts on HIV pathogenesis. Using genotype and gene expression data from the Gene Expression Omnibus repository and Genotype-Tissue Expression database, we observed cell-type-specific correlations between *CHD1L* and *PRKAB2* expression, with a strong positive association in whole blood and negative correlation in monocytes. Notably, we found that individuals with HIV set-point viral load associated variants exhibited significantly reduced *PRKAB2* expression in imputed whole blood models and *ex vivo* monocytes. Functional analyses using *PRKAB2* ^−/−^ induced pluripotent stem cells suggest that *PRKAB2* loss-of-function may influence *CHD1L* expression, and genes regulating cytokine activity, growth factor signaling, and pluripotency pathways associated with HIV infection. These results suggest that gene expression changes driven by HIV set-point viral load associated variants in the chromosome 1 impact multiple genes and, by influencing expression of *PRKAB2*, may result in altered expression of critical immune signaling processes. These findings advance our understanding of the contribution of host genetics on HIV pathogenesis and identifies new targets for *ex vivo* functional studies.

## Introduction

The HIV host-pathogen interaction is complex, with hundreds of human genes being implicated as candidate host restriction, inhibitory, proviral, or dependency factors (Hiatt et al., 2022). Recently, a genome-wide association study (GWAS) identified a novel region of chromosome 1 surrounding *PRKAB2*, *FMO5*, and *CHD1L* to be associated with reduced HIV set-point viral load (spVL) and functional follow-up identified *CHD1L* as a novel HIV inhibitory factor in U2OS and myeloid cells (McLaren et al., 2023). However, the potential of *PRKAB2*, the β2 subunit of the 5′AMP-activated protein kinase complex (AMPK), located upstream of *CHD1L*, was not evaluated for its effect on HIV infection.

AMPK is a heterotrimeric protein complex that is comprised of an alpha subunit (*PRKAA1/PRKAA2*), beta subunit (*PRKAB1/PRKAB2*), and gamma subunit (*PRKAG1/PRKAG2/PRKAG3*). This complex acts an essential regulator of cellular metabolism and during viral infection, functions as an inhibitory factor by reducing cholesterol, fatty acid, and protein synthesis of infected cells (Zhang and Wu, 2009; Zhang et al., 2012; Moreira et al., 2016). AMPK has also been shown to repress pro-inflammatory signaling (Jeong et al., 2009; Rutherford et al., 2016), activate anti-inflammatory signaling in macrophages (Sag et al., 2008), and has been shown to influence HIV pathogenesis (Bhutta et al., 2021). Therefore, we hypothesize that *PRKAB2* may directly or, through downstream signaling pathways, influence HIV infection.

The advent of large-scale siRNA and CRISPR-Cas9 screens have enabled researchers to screen thousands of genes as HIV factors, but knockdown and knockout screens have not previously implicated *CHD1L*, *PRKAB2*, or *FMO5* (Brass et al., 2008; Zhou et al., 2008; Yeung et al., 2009; Park et al., 2017). The lack of association suggests that these genes either do not function as HIV factors in the investigated cell types (HeLa, Jurkat T, or CEM-GXRCas9 cells) or may not act directly as HIV factors. In contrast, analysis of closely related genes to *PRKAB2,* such as *PRKAA1*, the dominant catalytic AMPKα subunit in myeloid cells (Sag et al., 2008), revealed its function as both an HIV host dependency factor (HDF) (Zhou et al., 2008) and an HIV inhibitory factor (Gélinas et al., 2018). These findings highlight that changes to specific AMPK subunits can affect HIV infection and supports investigating whether genetically regulated changes to *PRKAB2* expression are associated with HIV infection.

This study seeks to determine whether functional HIV spVL associated variants identified by the HIV spVL GWAS (McLaren et al., 2023) impact *PRKAB2* expression and downstream signaling pathways relevant to HIV infection. Using RNA-sequencing data from *PRKAB2* ^−/−^ immunopluripotent stem cells (iPSC), we assess whether *PRKAB2* affects *CHD1L* expression, downstream signalling pathways, and known HIV factors. The results of this study implicate *PRKAB2* as a potential HIV factor by identifying essential immune pathways altered by *PRKAB2* loss-of-function, providing additional insight into how the role of how the chromosome 1 region affects HIV infection.

## Methods

### Genotype data and variant imputation

The Immune Variation (ImmVar) project is a collaborative effort to characterize how genetic and environmental factors influence the immune response in healthy individuals (De Jager et al., 2015). Genotype data for 112 African American individuals from the ImmVar project was obtained from the database of Genotypes and Phenotypes (dbGaP; phs000815.v2.p1). Chromosome 1 variants were imputed for individuals from the ImmVar project using the 1000 Genomes reference panel with SHAPEIT (Delaneau et al., 2011) and IMPUTE2 (Howie et al., 2009). Variants with a score of <0.9 and Hardy-Weinberg equilibrium threshold of p < 1 × 10^−5^ were removed. Genotypes were assessed at rs59784663-A-G, rs72999655-A-G, rs7525622-G-A, and rs7300425-C-T. One individual was removed from subsequent analysis due to an incomplete genotype call at rs59784663-A-G.

### Gene expression databases

Gene expression profiles for individuals from the ImmVar project were evaluated to determine whether HIV spVL associated variants influence gene expression. Gene expression from naïve CD4^+^ T cells and monocytes, adjusted for batch effects, age, gender, and technical artifacts using principal components (PCs), were obtained from the Gene Expression Omnibus (GEO) (GSE56035). In brief, expression was profiled using Affymetrix GeneChip Human Gene ST 1.0 microarrays with background correction and normalization using the Robust Multichip Average method in Affymetrix Powertools as previously described (Raj et al., 2014). Sample, probe, and expression quality control, as well as the adjustment for non-genetic factors, including the regression of the top 14 PCs in monocytes and top 12 PCs in naïve CD4^+^ T cells, are detailed in Raj et al. (2014). In monocytes and naïve CD4^+^ T cells, the residuals are used to compare gene expression following correction for non-genetic factors.

Gene expression, in transcripts per kilo base million, were obtained from Genotype Tissue Expression (GTEx) database v8 in whole blood tissues for all available individuals (N = 756) (GTEx Consortium, 2020). The GTEx database v8 (N = 948) includes individuals of diverse ancestry, with 84.6%, 12.9%, 1.3%, and 1.1% of individuals identifying as white, African American, Asian, or unknown, although the specific ancestries for the 756 individuals were not available for this analysis. In this dataset, whole blood was defined as gathered from the femoral vein, subclavian vein, heart, and other sites (UBERON:0013756).

Raw RNA-seq reads and normalized RNA-seq read counts as transcripts per million were available for wild-type and *PRKAB2* ^−/−^ immunopluripotent stem cells (iPSCs) from the National Center for Biotechnology Information (NCBI) GEO database under the accession (GSE144043). In brief, NCBI generates RNA-seq count data using HISAT2 (Koga et al., 2019) to align sequences to GCA_000001405.15. Read counts were calculated using featureCounts with a 50% alignment threshold.

A summary of cohorts and data types accessed in this meta-analysis are available in Table 1.

**TABLE 1:** Summary of studies used in this meta-analysis.

Study cohort	Tissue	Sample number	Data type	Accession
Genotype-Tissue Expression Database V8	Whole blood	756 identified as white, African American, Asian, or unknown	RNA-Sequencing	www.gtexportal.org
ImmVar project	N/A	N = 110 African Americans	Genotypes	phs000815.v2.p1
	Monocytes	N = 110 African Americans	Microarray Expression	GSE56033
	Naïve CD4^+^ T cells	N = 110 African Americans	Microarray Expression	GSE56034
Ziegler et al. (2020)	Human iPSCs	N = 12	RNA-sequencing	GSE144043
McLaren et al. (2023)	N/A	3,886 individuals of African ancestry	Genotypes	N/A
Kachuri et al. (2023)	Whole Blood	757 African Americans	Whole-blood PrediXcan models	N/A

### Correlation of CHD1L and PRKAB2 gene expression

Previous studies have suggested that HIV spVL associated variants in the chromosome 1 region regulate *CHD1L* expression in a cell-type-specific manner (McLaren et al., 2023). Therefore, we sought to investigate genetically regulated *PRKAB2* expression across multiple immune cell types. Gene expression data was compared for whole blood samples from GTEx as transcripts per million (TPM) or monocytes and naïve CD4^+^ T cells from the ImmVar project as residuals following control for non-genetic factors. For each dataset, a two-sided Pearson correlation was used to determine correlation between *CHD1L* and *PRKAB2*, with p < 0.05 defined as statistically significant. A linear regression model, along with a 95% confidence interval, was to visualize the magnitude and direction of correlation between *PRKAB2* and *CHD1L* expression. Statistical analysis was performed using R (v4.1.2).

### Imputation of CHD1L expression from whole blood models

Genomic data from 3,886 individuals of African ancestry were obtained from ten independent GWAS under the International Collaboration for the Genomics of HIV (ICGH) (N = 3,886), as previously described (McLaren et al., 2023). Predictive gene expression models trained on African American whole blood eQTLs from the Genes-environments and Admixture in Latino Americans (GALA II) and the Study of African Americans, Asthma, Genes, and Environments (SAGE) cohorts were applied to the 3,886 individual-level genotypes using PrediXcan (Gamazon et al., 2015). Imputed *CHD1L* expression was compared amongst individuals with allelic combinations of functional HIV spVL associated variants: rs72999655-A-G, rs7525622-G-A, and rs73004025-C-T from the ICGH cohort. Statistical significance was determined using a Wilcoxon Rank Sum Test at p < 0.05.

### RNA-sequencing and pathway analysis

Given that GWAS variants typically exert a small effect on gene expression changes, we sought to determine whether *PRKAB2* was associated with immune signaling pathways using expression data from *PRKAB2* knockout cells to prioritize subsequent association testing. Differential gene expression analyses of *PRKAB2* and *CHD1L* expression were performed using NCBI generated TPM from wild-type iPSCs (N = 4), *PRKAB2* ^*−/−*^ clone one (C1; N = 4), and *PRKAB2* ^−/−^ clone two (C2; N = 4) from the GEO database (GSE144043). Significant differences determined using a Wilcoxon Rank Sum Exact Test against p < 0.05.

Transcriptome differences between wild-type iPSCs, *PRKAB2* ^*−/−*^ C1, and *PRKAB2* ^*−/−*^ C2, were assessed using NCBI generated RNA-seq counts from the GEO database (GSE144043). Lowly expressed genes were filtered based on a minimum count threshold of at least 10 in a given sample. Differential expression was performed using DESeq2 using FDR <0.05, α = 0.05 as statistically significant. Visualization and statistical analysis were performed using R (v4.1.2).

The Database for Annotation, Visualization and Integrated Discovery (DAVID; version v2023q4) was used to perform pathway analysis and functional annotation of DEGs (Huang et al., 2009; Sherman et al., 2022). Functional enrichment was classified for Gene Ontology, Reactome, and KEGG pathway terms using medium classification stringency and an enrichment score of greater than two. Individual annotations were significant with an FDR cut-off threshold of 0.05. HIV interactions were characterized by any overlap of genes with the HIV interaction category.

## Results

### PRKAB2 and CHD1L exhibit cell-type-specific correlation patterns

**FIGURE 1:**
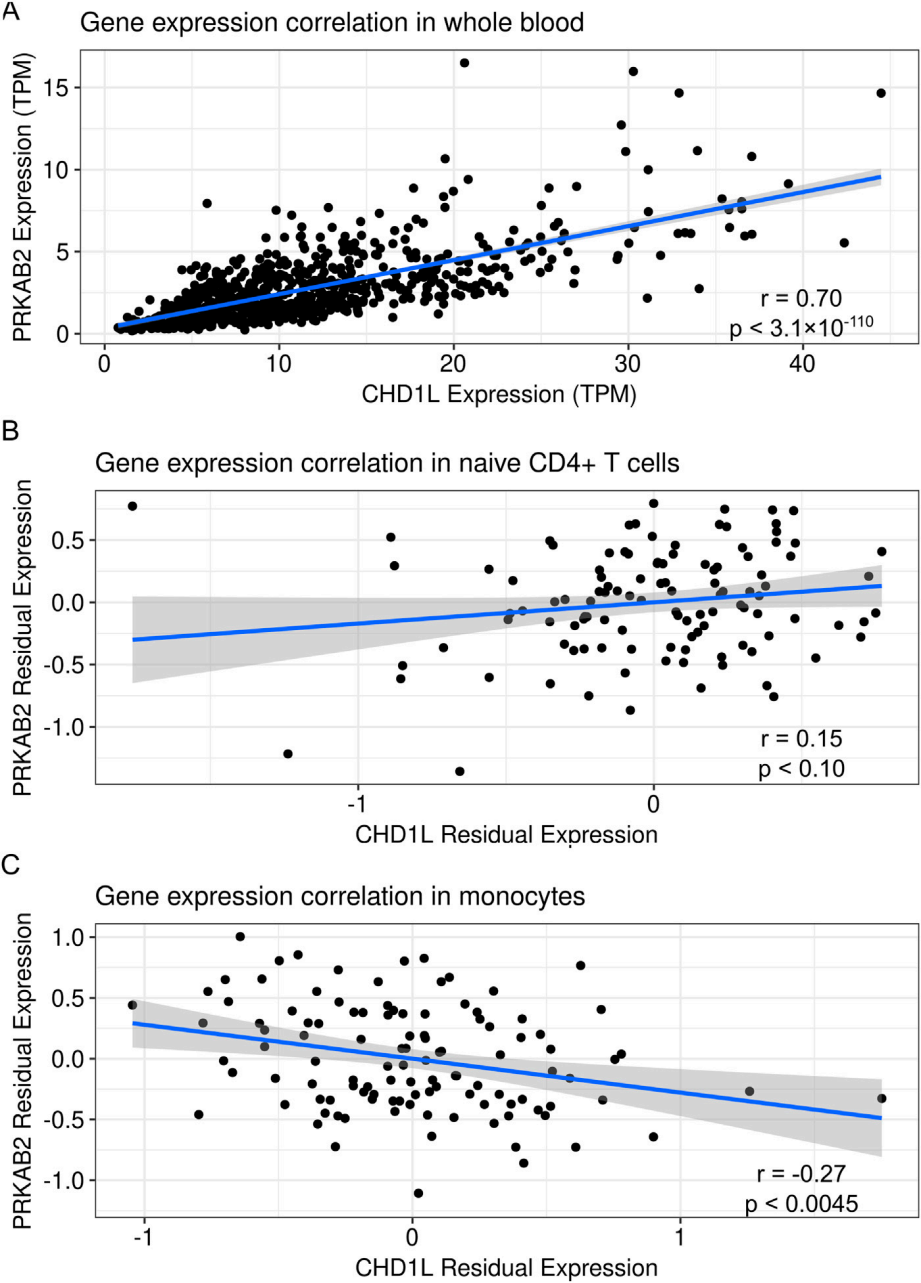
Correlation of *CHD1L* and *PRKAB2* expression in human blood tissues. **(A)** Transcripts per million for *CHD1L* and *PRKAB2* expression from whole blood in the Genotype-Tissue Expression database (N = 756). **(B)** Residuals of *PRKAB2* and *CHD1L* expression after controlling for batch, sex, and technical variation in naïve CD4^+^ T cells. **(C)** Residuals of *PRKAB2* and *CHD1L* expression after controlling for batch, sex, and technical variation in monocytes. The blue line represents a linear regression of the values surrounded by standard error in grey.

Regulatory variants can influence the expression of multiple genes which collectively contribute to complex phenotypes (Ying et al., 2023). While previous studies have focused on genetically regulated *CHD1L* expression (McLaren et al., 2023), we hypothesized that *PRKAB2* may be co-regulated due to its proximity to *CHD1L*. To address this, we analyzed the correlation between *PRKAB2* and *CHD1L* expression in immune cells from the GTEx and ImmVar databases. Gene expression from the GTEx datasets showed a strong positive correlation between *PRKAB2* and *CHD1L* expression from whole blood tissues (r = 0.7, p < 3.1 × 10^−110^) (Figure 1A). However, we observed no significant correlation in naïve CD4^+^ T cells (r = 0.17, p < 0.081) (Figure 1B) but a significant negative correlation in monocytes (r = −0.29, p < 0.002) (Figure 1C). These correlations suggest that *CHD1L* and *PRKAB2* likely share regulatory features in immune cells but the magnitude and direction of effect may be cell-type-specific.

### HIV spVL decreasing variants in the chromosome 1 region are associated with decreased PRKAB2 expression

**FIGURE 2:**
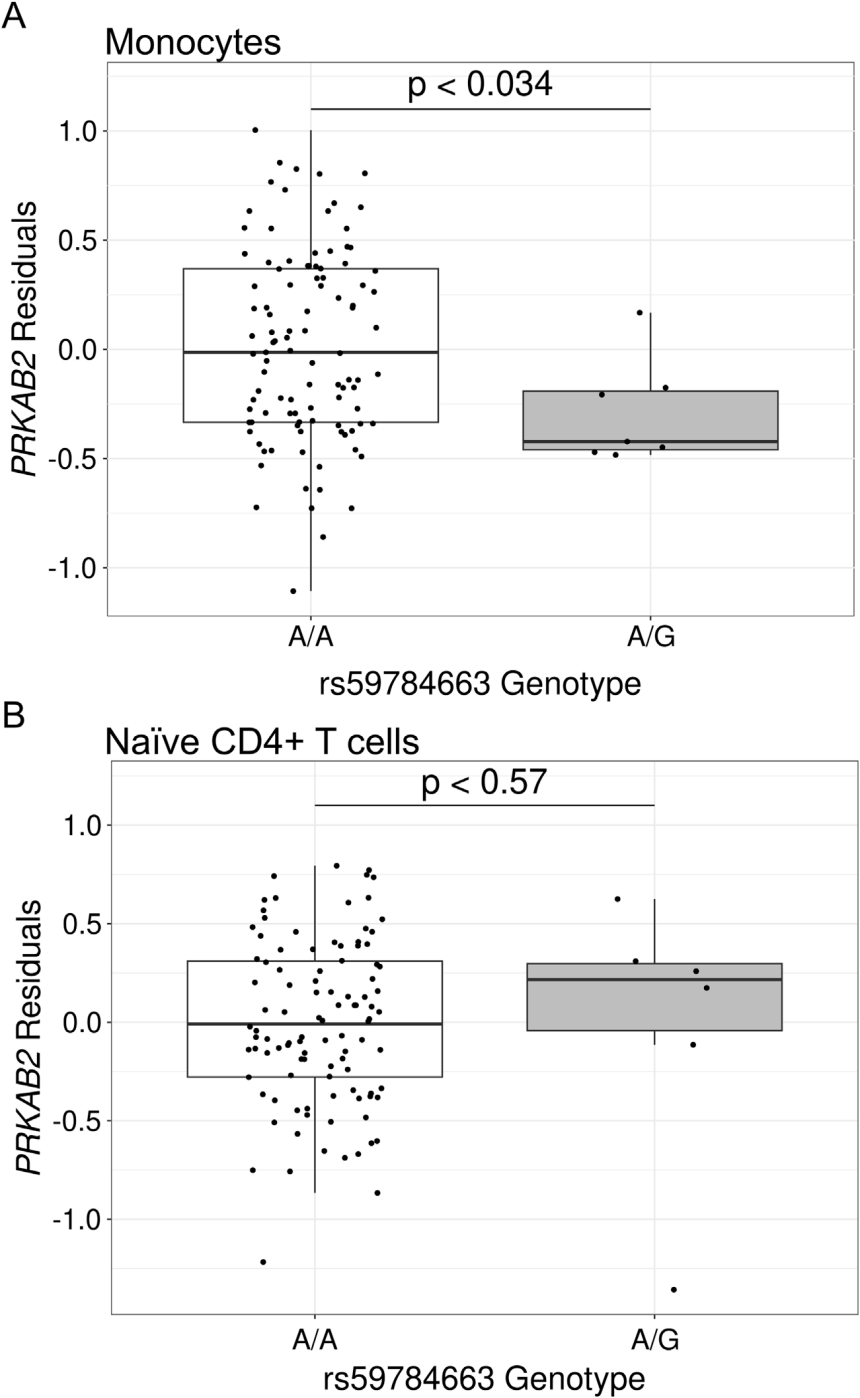
Gene expression of *PRKAB2* based on rs59784663-A-G genotype in African populations. Gene expression is the residuals following control for non-genetic factors in African populations for **(A)** monocytes or **(B)** naïve CD4^+^ T cells. Significance was determined using a two-sided Wilcoxon Rank Sum Test.

We next assessed the impact of HIV spVL associated variants on *PRKAB2* expression to determine whether regulatory features in the chromosome 1 region influence both genes. In monocytes, individuals heterozygous for lead GWAS variant rs59784663-A-G (N = 6), had a significant reduction of *PRKAB2* expression compared to homozygous reference individuals (N = 103; p < 0.036) (Figure 2A) but no significant effect in naïve CD4^+^ T cells (p < 0.56) (Figure 2B).

**FIGURE 3:**
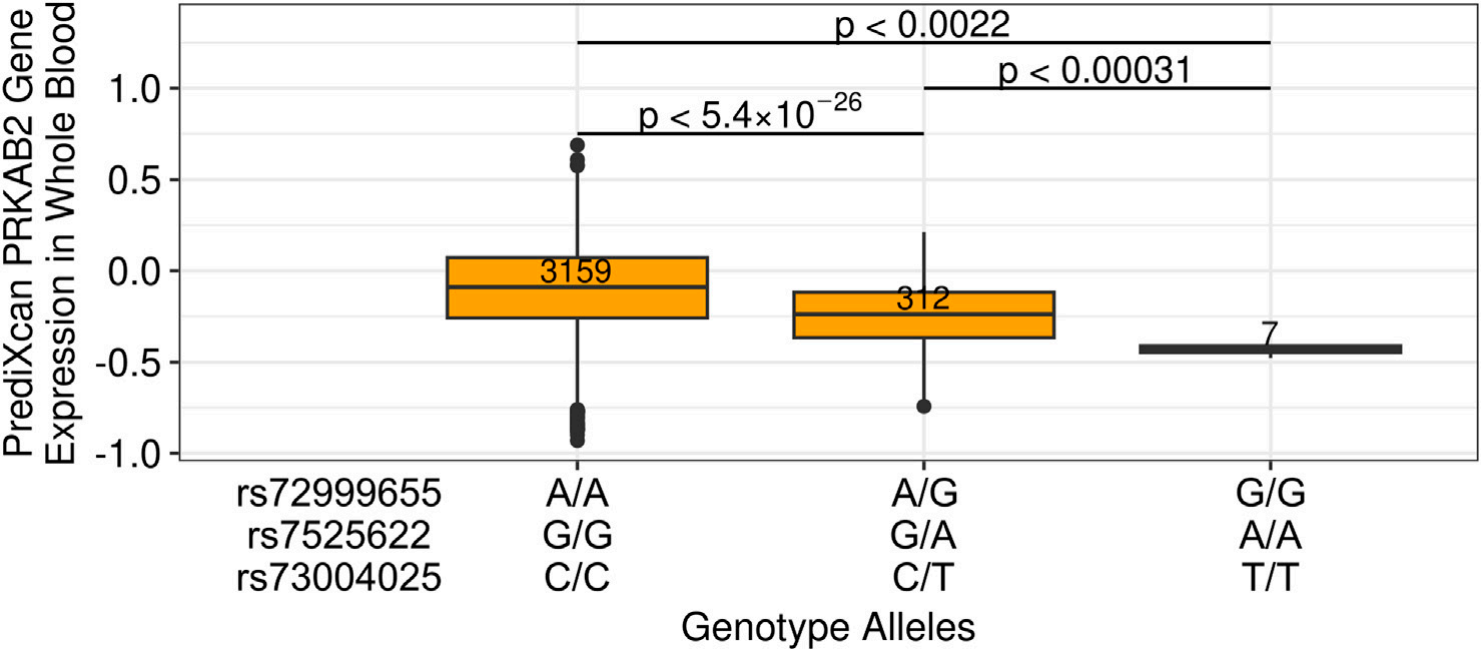
Imputed *PRKAB2* expression changes based on alleles of HIV spVL associated genetic variants. *PRKAB2* expression was generated using whole blood PrediXcan models from the Genes-Environments and Admixture in Latino Asthmatics and the Study of African Americans, Asthma, Genes, and Environments cohorts. Allelic combinations are shown for individuals homozygous reference, heterozygous, or homozygous alternate at all variants.

We then sought to determine the effect of candidate causal variants rs72999655-A-G, rs7525622-G-A, and rs73004025-C-T on *PRKAB2* expression, and to have sufficient sample size for analysis, imputed *PRKAB2* expression from whole blood models using 3,886 individual-level genotypes from the ICGH cohort (McLaren et al., 2023). Individuals heterozygous for rs72999655-A-G, rs7525622-G-A, and rs73004025-C-T (N = 312) exhibited lower *PRKAB2* expression than homozygous reference (N = 3,159; p < 1.65 × 10^−26^) and homozygous alternate individuals (N = 7) exhibited much lower *PRKAB2* expression than homozygous reference (p < 0.00031) (Figure 3). Overall, these results show that HIV spVL associated variants likely influence *PRKAB2* expression and highlights the need to investigate potential downstream consequences of HIV infection.

### PRKAB2^−/−^ iPSC models suggest PRKAB2 does not strongly regulate CHD1L expression

**FIGURE 4:**
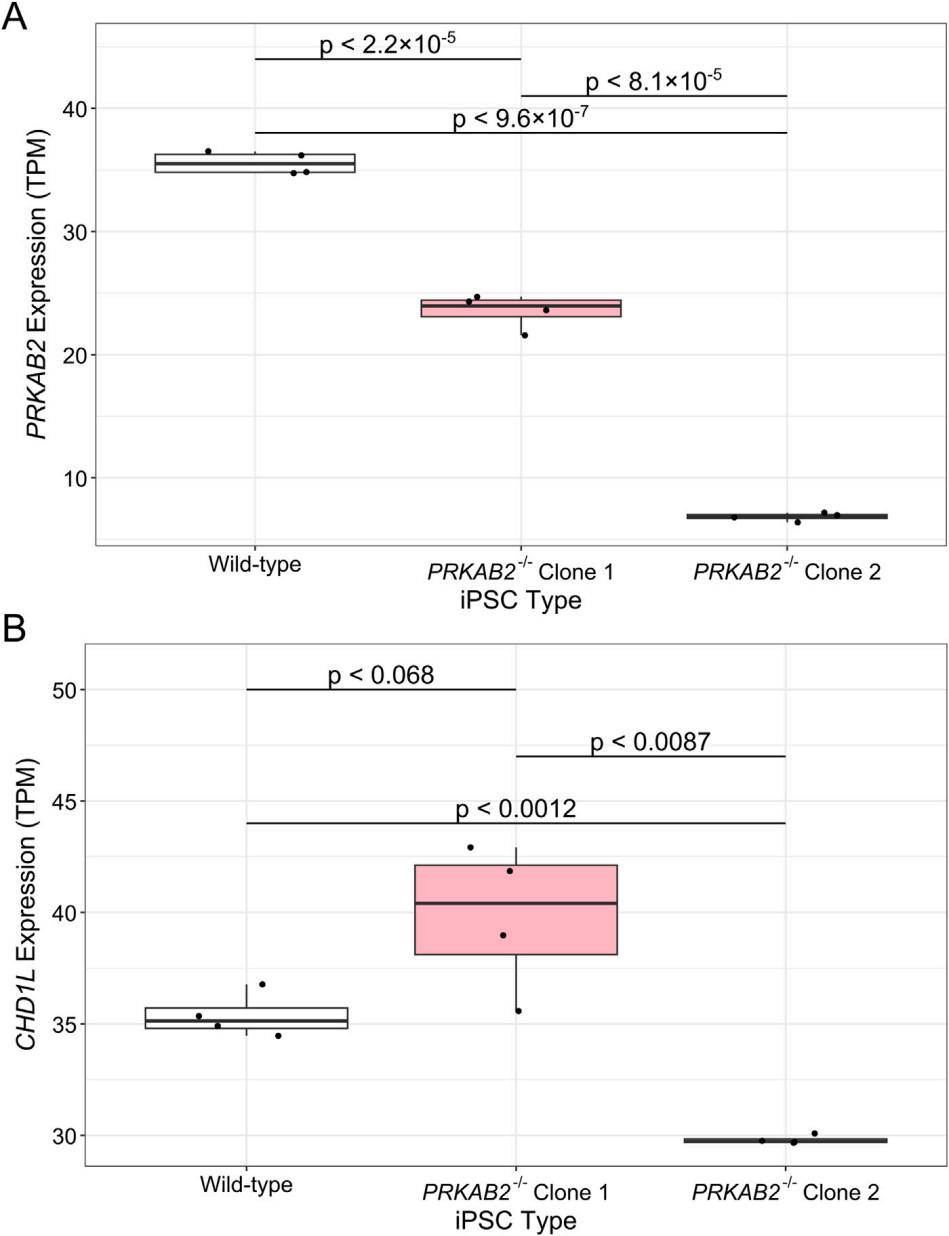
Gene expression differences in PRKAB2^−/−^ loss-of-function iPSCs. Gene expression of **(A)** *PRKAB2* and **(B)** *CHD1L* in wild-type iPSCs, *PRKAB2* knockout clone 1 iPSCs, and *PRKAB2* knockout clone 2 iPSCs. Significance was determined using a Wilcoxon Rank Sum Exact Test.

Further investigation into the relationship between *PRKAB2* and *CHD1L* was performed using GEO gene expression data from *PRKAB2* ^−/−^ iPSC models (GSE144043) (Ziegler et al., 2020). In PRKAB2^*−/−*^ iPSCs, *PRKAB2* expression was significant reduced compared to wild-type controls (p < 2.2 × 10^−5^ and p < 9.6 × 10^−7^ for clones C1 and C2, respectively), while a larger reduction was observed between C2 compared to C1 (p < 8.1 × 10^−5^) (Figure 4A). However, despite reduced *PRKAB2* expression in both clones, *CHD1L* expression was inconsistent. While *CHD1L* expression was not significantly different between wild-type and C1 iPSCs (p < 0.068), we observed higher *CHD1L* expression in C1 clones, but a significant reduction of *CHD1L* expression observed in C2 compared to wild-type (p < 0.0012) (Figure 4B). The variability between clones may reflect differences in gRNA efficacy or off-target effects during generation of *PRKAB2* ^*−/−*^ knockouts. While the data is unclear in the potential of AMPKβ2-mediated *CHD1L* expression, AMPK is known to activate DNA repair pathways (Szewczuk et al., 2020), suggesting that AMPKβ2 may alter expression of *CHD1L* only under specific physiological conditions.

### Chromosome 1 variants are associated with downstream gene expression differences

One of the major challenges in identifying changes to *PRKAB2* signaling pathways is that the functional AMPKβ2 complex regulates expression of downstream genes through phosphorylation, which is not detectable through RNA-sequencing analyses. However, AMPK regulates the activation of transcription factors (Cantó and Auwerx, 2010), indicating that signalling pathways downstream of *PRKAB2* may be differentially expressed. Therefore, we leveraged gene expression profiles from the wild-type and *PRKAB2* ^*−/−*^ iPSCs (GSE144043) to determine potential pathways influence by *PRKAB2*.

We first sought to determine whether *PRKAB2* ^−/−^ C1 and C2 exhibited different gene expression profiles given the differences in *PRKAB2* expression observed previously. We observed 58 differentially expressed genes (DEGs) between C1 and C2 suggesting that differences in gRNA efficiency or off-target effects may influence gene expression profiles. Additionally, when comparing DEGs in C2 and wild-type iPSCs, the most significant DEG was *PRKAB2* (log_2_FC = −2.4, FDR < 2.2 × 10^−308^), but when comparing C1 and wild-type iPSCs, *PRKAB2* ranked 44th (log_2_FC = −0.70, FDR < 7.9 × 10^−59^). Therefore, we chose to analyze gene expression differences between wild-type and *PRKAB2* ^−/−^ C2, given that C2 exhibited a larger reduction in *PRKAB2* expression compared to C1.

**FIGURE 5:**
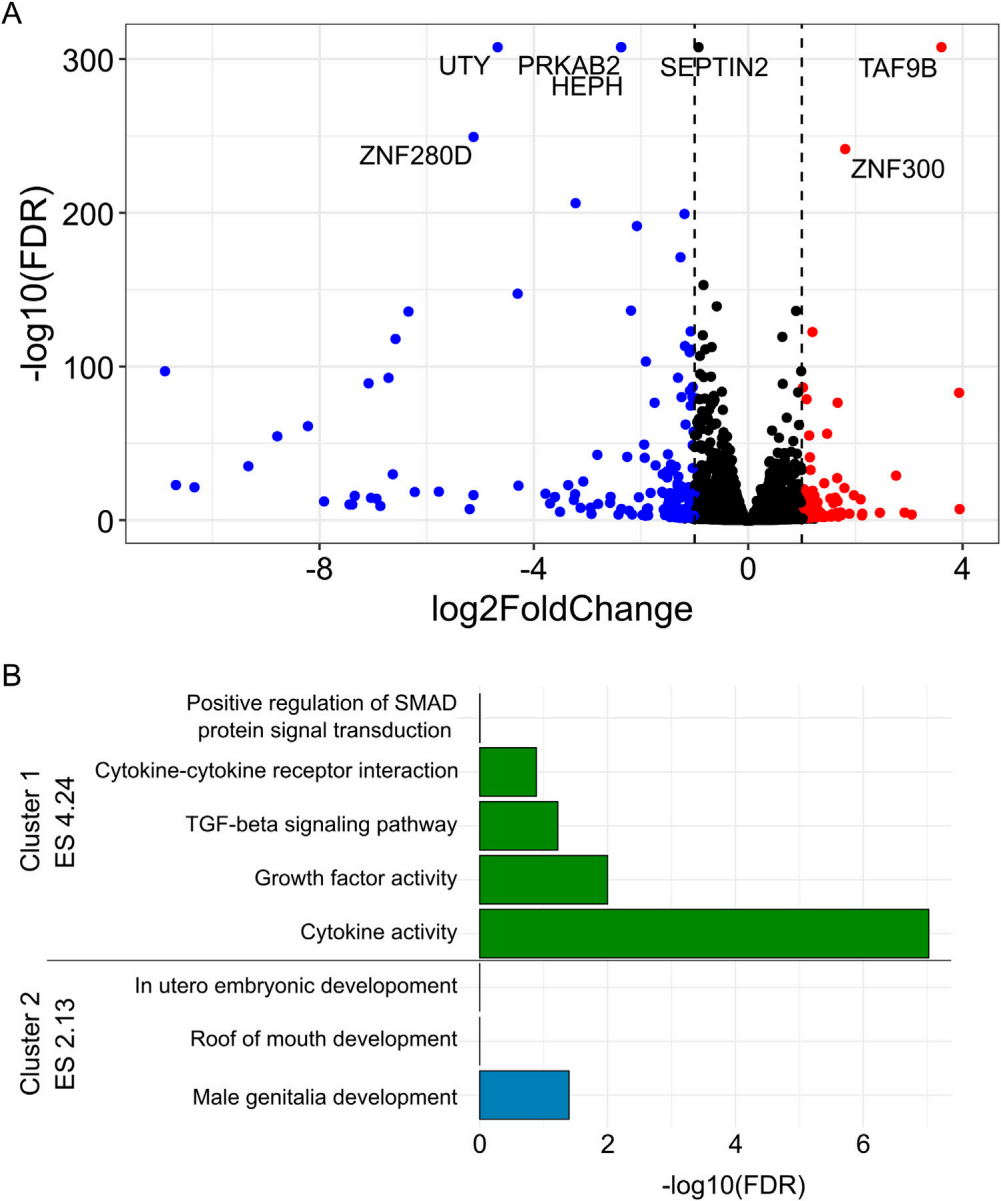
Differentially expressed genes between PRKAB2^−/−^ knockout and wild-type iPSCs. **(A)** (Blue) Significant downregulated genes (FDR < 0.05, log_2_ fold change < 1) in *PRKAB2* knockout clone two cells. (Red) Significant upregulated genes (FDR < 0.05, log_2_ fold change > 1) in *PRKAB2* knockout clone two cells. **(B)** Enrichment clustering of differentially expressed genes revealed two distinct clusters with an enrichment score of greater than two.

There were 170 downregulated and 145 upregulated genes in *PRKAB2* ^*−/−*^ C2 compared to wild-type iPSCs (FDR < 0.05, |log2 fold change| ≥ 1, Figure 5A). These genes were significantly enriched for functions related to cytokine activity (17 genes, FDR < 9.5 × 10^−8^) and growth factor activity (10 genes, FDR <0.012), as well as KEGG pathways regulating stem cell pluripotency (9 genes, FDR <0.028; Table 2). Clustering analysis revealed two major groups: one associated with cytokine activity (enrichment score 4.24) and the other with male genitalia development (enrichment score 2.13; Figure 5B). Given the relevance of cytokine activity to HIV infection, we prioritized this group for further analysis. Among the differentially expressed genes in the cytokine activity pathway, *TNFSF9* (log_2_FC = −1.5, FDR < 1.8 × 10^−32^)*, TGFB2* (log_2_FC = −1.0, FDR < 0.000071)*,* and *SCG2* (log_2_FC = 1.003, FDR < 0.0069), stood out as known HIV interactors using DAVID annotation.

**TABLE 2:** DAVID enrichment of differentially expressed genes in gene ontology and KEGG categories.

Category	Term	Count	%	FDR
Goterm Mf Direct	cytokine activity	17	5.56	9.45 × 10^−8^
Goterm Cc Direct	extracellular space	42	13.73	0.0093
Goterm Mf Direct	growth factor activity	10	3.27	0.012
Kegg Pathway	Signaling pathways regulating pluripotency of stem cells	9	2.941	0.029
Goterm Bp Direct	cell fate commitment	7	2.28	0.038
Goterm Bp Direct	male genitalia development	5	1.63	0.038

However, given that *PRKAB2* ^−/−^ C1 and C2 exhibited significant differences in both *PRKAB2* and *CHD1L* expression, we replicated the transcriptomic analysis comparing wild-type iPSCs to both *PRKAB2* ^*−/−*^ C1 and C2, together. This resulted in 125 downregulated and 160 upregulated genes in *PRKAB2* ^−/−^ knockout iPSCs compared to wild-type iPSCs, but the most significant enriched pathway remained cytokine activity (Supplementary Figure S1). Collectively, these results show that individuals with HIV spVL associated variants exhibit reduced *PRKAB2* expression and reduced *PRKAB2* expression alters cytokine regulation, suggesting that the chromosome 1 region may regulate HIV spVL through these immune pathways.

## Discussion

During the analysis of a recent GWAS of HIV spVL, *CHD1L* was identified to be the candidate causal gene and implicated as a novel HIV inhibitory factor (McLaren et al., 2023). However, the question remains as to why *CHD1L* was not detected in previous screens of HIV inhibitory factors (Gélinas et al., 2018). While cell-type-specific effects may explain some variation, it is also likely that multiple chromosome 1 genes may collectively contribute to control HIV infection through downstream signaling pathways (Broekema et al., 2020). Therefore, we chose to investigate the role of *PRKAB2*, part of the AMPKβ2 complex, as AMPK has been previously associated with HIV infection (Zhou et al., 2008; Zhang and Wu, 2009).

Our findings indicate that *PRKAB2* and *CHD1L* expression is positively correlated in whole blood but negatively correlated in monocytes, supporting cell-type-specific gene regulation. Previously fine-mapping of a HIV spVL GWAS identified three candidate causal GWAS variants, rs72999655-A-G, rs7525622-G-A, and rs73004025-C-T, and that alleles linked to lower HIV spVL were associated with increased *CHD1L* expression in monocytes (Tough et al., 2025). In this study, individuals heterozygous for rs72999655-A-G, rs7525622-G-A, and rs73004025-C-T exhibited reduced *PRKAB2* expression in monocytes and whole blood, but no differences in naïve CD4^+^ T cells. While the biological mechanism how these variants drive gene expression changes remains unclear, these results suggest that lower *PRKAB2* expression is associated with reduced HIV spVL.

Next, we hypothesized that *PRKAB2* may directly influence *CHD1L* expression, as AMPK activation initiates DNA repair pathways involving CHD1L (Wu et al., 2013; Szewczuk et al., 2020). We observed that *PRKAB2* loss-of-function had no effect on *CHD1L* expression in *PRKAB2* ^*−/−*^ C1 but resulted in significant downregulation in *PRKAB2* ^−/−^ C2. While both C1 and C2 were identified to have complete protein ablation (Ziegler et al., 2020), it is unclear whether the observed differences were driven by off-target effects, environmental, or physiological conditions. To address this ambiguity, additional studies are necessary to investigate whether AMPKβ2 influences *CHD1L* expression under conditions of AMPK activation such as DNA damage, low cellular metabolism, or HIV infection.

While our previous experiments showed that individuals with protective HIV spVL associated variants have reduced *PRKAB2* expression, the overall impact on downstream signaling pathways is unknown. This is in part because AMPKβ1 and AMPKβ2 exhibit different transcriptome profiles (Ziegler et al., 2020), but specific AMPKβ2 pathways associated with HIV infection are unclear. To address this, we compared the transcriptome profiles from *PRKAB2* ^*−/−*^ and wild-type iPSCs, which identified the most significant pathway altered by *PRKAB2* loss-of-function was regulation of cytokine signaling. Cytokine and chemokine levels, prior to HIV infection, can be strong predictors of disease progression (Huang et al., 2016; Ngcobo et al., 2022) and increased AMPK activity is associated with suppression of pro-inflammatory signaling in macrophages (Sag et al., 2008). Therefore, this provides evidence that HIV spVL associated variants in the chromosome 1 region likely influence HIV spVL through changes to cytokine signaling and immune activation pathways.

Three genes regulating cytokine activity were identified by the DAVID analysis to interact with HIV proteins: *TNFSF9, TGFB2*, and *SCG2*. The gene *SCG2*, upregulated in *PRKAB2* ^*−/−*^ *,* plays an important role in regulating the immune response through the assembly and function of the major histocompatibility complex, PI3K-Akt, TGF-β, and JAK-STAT pathways (Wang et al., 2021; Steinfass et al., 2023). The gene *TNFSF9*, also known as 4-1BBL and downregulated in *PRKAB2* ^*−/−*^, produces a pro-inflammatory immune response and a key component of cytotoxic CD8^+^ T cells, macrophages, and monocytes activation (Ju et al., 2003; Wang et al., 2007; Olofsson et al., 2008). The gene *TGFB2,* downregulated in *PRKAB2* ^*−/−*^ *,* has immunosuppressive functions and regulates activation of dendritic and T cells (Tu et al., 2020). Collectively, these genes implicate additional pathways downstream of AMPK signaling associated with HIV pathogenesis and identifies targets for future research to elucidate mechanisms by which HIV spVL associated variants influence HIV spVL.

However, there are limitations to this study that should be acknowledged. Firstly, the cohort data analyzed here consists of individuals of African American ancestry from the ImmVar and GALA II/SAGE cohorts and requires replication in African cohorts to ensure broader generalizability. Secondly, our analysis of differentially expressed genes utilized unstimulated iPSCs, whereas AMPK is typically activated in response to metabolic stress (Steinberg and Hardie, 2023). While this provides insight into pathways associated with AMPKβ2, future studies should assess the function in primary immune cells and during HIV infection.

Overall, this study highlights that HIV spVL associated variants influence *PRKAB2* expression and raises important questions about whether this effect is synergistic, antagonistic, or independent of *CHD1L* regulation. Furthermore, as AMPK is a regulator of immune function, future studies should investigate whether chromosome 1 genes differentially affect HIV pathogenesis during acute and chronic infection, providing insights into their potential as therapeutic targets.

## Data Availability

The original contributions presented in the study are included in the article/Supplementary Material, further inquiries can be directed to the corresponding authors.

## Supplementary material

SUPPLEMENTARY FIGURE S1 Differential gene expression profiles between wild-type iPSCs and *PRKAB2* ^−/−^ clones (C1 and C2). **(A)** (Blue) Significant downregulated genes (FDR <0.05, log_2_ fold change <1) in *PRKAB2* knockout C1 and C2 compared to wild-type iPSCs. (Red) Significant upregulated genes (FDR <0.05, log_2_ fold change >1) in *PRKAB2* knockout C1 and C2 compared to wild-type iPSCs. **(B)** Enrichment of DAVID terms amongst significant differentially expressed genes.
