# Supplementary figures and images for: Chromosome 1 variants associated with decreased HIV set-point viral load correlate with PRKAB2 expression changes
Source: Front Genet. 2025 Mar 6;16:1551171. doi: 10.3389/fgene.2025.1551171 (PMC11922826; doi:10.3389/fgene.2025.1551171)

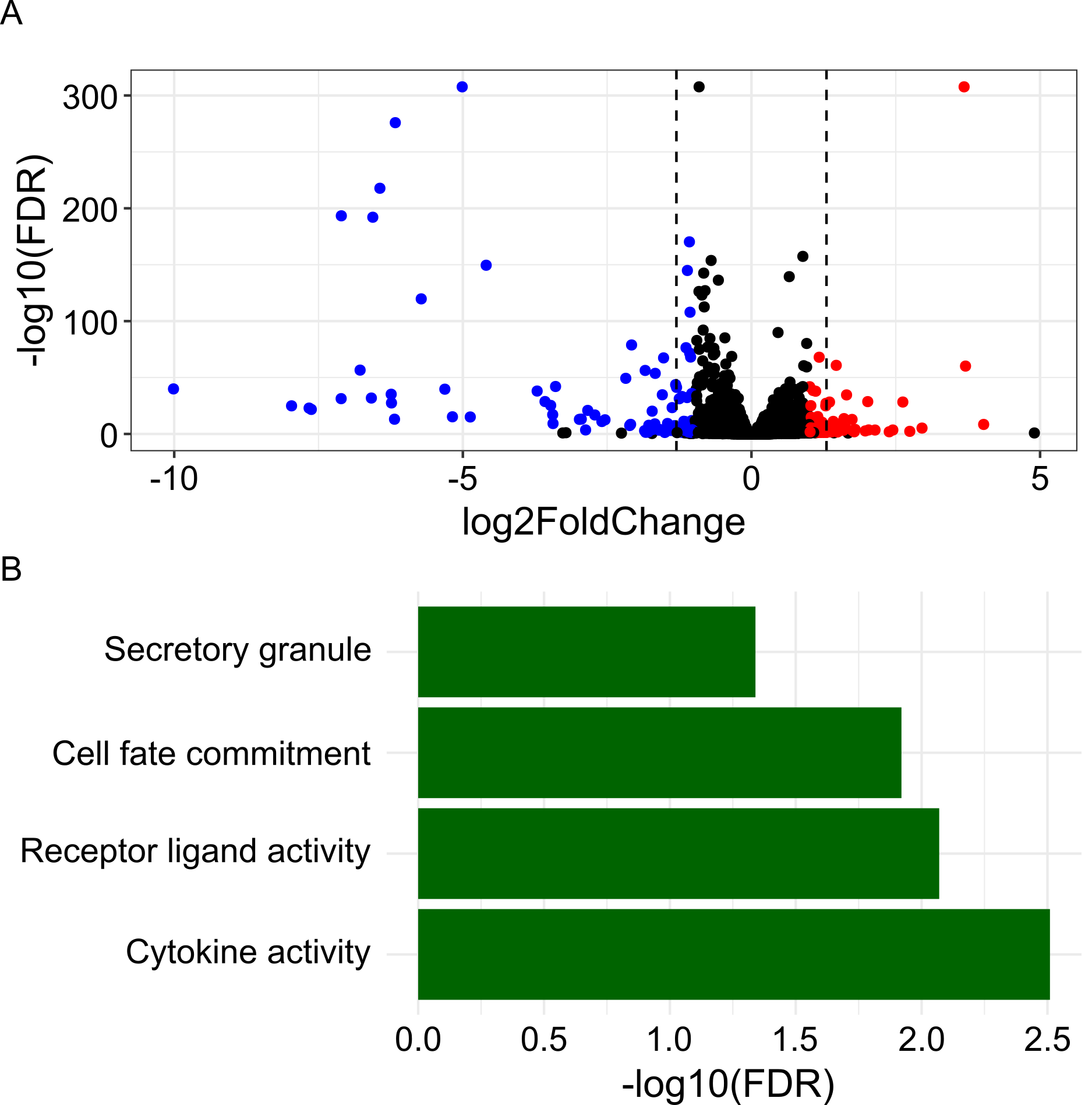

Supplement: Supplementary file 1 [file Image1.png]
